# A Personalised Pacing and Active Rest Rehabilitation Programme for Post-Exertional Symptom Exacerbation and Health Status in Long COVID (PACELOC): A Prospective Cohort Study

**DOI:** 10.3390/jcm14010097

**Published:** 2024-12-27

**Authors:** Belinda Godfrey, Jenna Shardha, Sharon Witton, Rochelle Bodey, Rachel Tarrant, Darren C. Greenwood, Manoj Sivan

**Affiliations:** 1 Leeds Long COVID Community Rehabilitation Service, Leeds Community Healthcare NHS Trust, Leeds LS11 0DL, UK; belindagodfrey@hotmail.co.uk (B.G.); jenna.shardha1@nhs.net (J.S.); sharon.witton3@nhs.net (S.W.); rochellebodey@nhs.net (R.B.); rachel.tarrant1@nhs.net (R.T.); 2 Leeds Institute of Cardiovascular and Metabolic Medicine, University of Leeds, Leeds LS2 9JT, UK; d.c.greenwood@leeds.ac.uk; 3 Leeds Institute for Data Analytics, University of Leeds, Leeds LS2 9JT, UK; 4 National Demonstration Centre of Rehabilitation Medicine, Leeds Teaching Hospitals NHS Trust, Leeds LS7 4SA, UK; 5 Academic Department of Rehabilitation Medicine, Leeds Institute of Rheumatic and Musculoskeletal, Medicine, University of Leeds, Leeds LS1 3EX, UK

**Keywords:** long COVID, post-COVID-19 syndrome, COVID-19, post-exertional symptom exacerbation, pacing, rehabilitation

## Abstract

**Background:** Post-COVID-19 Syndrome or long COVID (LC) is a novel public health crisis and, when persistent (>2 years), is a long-term condition. Post-exertional symptom exacerbation (PESE) is a characteristic symptom of LC and can be improved in a structured pacing rehabilitation programme. **Aims:** To evaluate the effect of an 8-week structured World Health Organisation (WHO) Borg CR-10 pacing protocol on PESE episodes, LC symptoms, and quality of life in a cohort of individuals with long-term LC. **Methods:** Participants received weekly telephone calls with a clinician to discuss their activity phase, considering their PESE symptoms that week. They completed the Leeds PESE questionnaire (LPQ), C19-YRS (Yorkshire Rehabilitation Scale), and EQ-5D-5L at the beginning of the programme (0 weeks), the end of programme (8 weeks), and at final follow-up (12 weeks). **Results:** Thirty-one participants (duration of LC symptoms: 29 months) completed the programme. The PESE episodes decreased in number each week (15% fewer each week, 95% CI: 11% to 20%, *p* < 0.001) and were of shorter duration and milder severity each week. The changes in C19YRS symptom severity and functional disability (0–12 weeks) were statistically significant but not clinically significant. The EQ5D-5L index score change was not statistically significant. **Conclusions:** A structured pacing protocol effectively reduced PESE episode frequency, duration, and severity but did not produce clinically significant changes in LC symptoms, reflecting the long-term nature of the condition in this cohort.

## 1. Introduction

It has now been over four years since COVID-19 became a global pandemic and we saw the first signs of post-COVID-19 Syndrome (PCS) or long COVID (LC). Defined as signs and symptoms that develop during or after an infection consistent with COVID-19, which continue for more than 12 weeks and are not explained by an alternative diagnosis [1], LC continues to affect millions of people worldwide. Office for National Statistics (ONS) data from March 2023 estimated that 1.9 million people in the UK had self-reported LC, with those in the 35–69 age group reporting symptoms most frequently [2] A further ONS study from the winter of 2023–24 reported the figure for England and Scotland as 2 million, or 3.3% of the population. Of those people, 71% reported having had symptoms for at least one year, 51% at least two years, and 31% at least three years. A total 74.7% of respondents reported an adverse impact on day-to-day activities and 19.2% stated that their day-to-day activities were ‘limited a lot’ [3].

Of the many symptoms reported, post-exertional symptom exacerbation (PESE), or post-exertional malaise (PEM), has emerged as one of the most common and debilitating, with 86% of respondents to a TUC survey in 2022 reporting it as a feature of their LC [4]. PESE is also a defining feature of myalgic encephalomyelitis/chronic fatigue syndrome (ME/CFS); therefore, much of what we know comes from the literature associated with this condition. PESE is characterised by worsening fatigue, pain, cognitive function, and other symptoms in response to any form of exertion/activity [5,6,7]. Symptoms can start soon after activity or have a delayed onset of up to 72 h, lasting for days, weeks, or longer [8,9,10,11]. Symptom severity is disproportionate to the perceived level of exertion, with both the severity and behaviour of symptoms being variable between patients and indeed within the same patient [12]. This makes PESE difficult to predict and manage, creating challenges for patients and clinicians alike given the already fluctuating nature of LC symptoms. For some, fear of an exacerbation becomes so great that they avoid activity wherever possible, risking isolation, deconditioning, and declining overall health. Due to the numbers affected by LC and the prevalence of PESE within the condition, there is a growing disability burden amongst patients and a potentially devastating effect on their roles at home, work, and in communities. The associated impact on healthcare, economies, and society is of great concern due to the vast number of working-age people affected by LC and the numbers continuing to report a lack of full recovery, to the point that in some, it has become a long-term condition [13,14,15,16,17].

There is currently no medical/pharmacological treatment for PESE; therefore, effective symptom management strategies are needed [9,11,18]. Strategies for PESE management generally involve pacing, which should not be confused with traditional graded exercise as this can be detrimental to those experiencing PESE [19]. Effective pacing encourages patients to be as active as possible within the limits of their symptoms, also known as their energy envelope [20,21]. It aims to enable manageable and consistent levels of activity rather than ‘boom–bust’ patterns, whereby patients push themselves to continue despite fatigue and other symptoms, only to trigger debilitating ‘crashes’ [5,18,22,23]. Currently, there is a lack of consensus on how to pace effectively; therefore, more work is required [24]. A scoping review of pacing in 2023 found a lack of studies, especially in the years before the pandemic, and that the quality of research was generally low to moderate. Many studies gathered patient opinions regarding pacing, rather than actually implementing it, and the use of patient-reported outcome measures (PROMS) was low. Effective pacing methods involved activity planning, consistency, energy management techniques, and avoiding activity progression, but the authors concluded that the low quality and quantity of research indicated that further work is urgently needed [5].

The World Health Organisation Borg CR-10 (WHO Borg CR-10) pacing algorithm has five phases of activity to monitor and adjust exertion levels. Users are encouraged to use the algorithm as a reference tool, matching the phase of activity to current functional ability, only progressing to the next phase if they achieve a PESE-free period and reverting to an easier phase during times of symptom exacerbation [25]. We, in our previous study, tested a 6-week structured WHO Borg CR-10 pacing protocol, the results of which showed a significant reduction in PESE episodes and improved quality of life in a cohort of LC patients [25]. However, clinicians in the service had observed that patients often struggled with the notion of rest. Many were not prioritising it and were unsuccessfully using sleep as a strategy for PESE. The pacing protocol required refinement to both promote and include guidance on active rest, and it needed to be tested over a slightly longer period than our previous study (i.e., 8 weeks). We were particularly interested in testing the protocol in persistent LC (>2 years) when LC is a long-term condition (LTC). The aims of this study were to test the modified protocol in a cohort of persistent LC patients.

## 2. Materials and Methods

Patients over the age of 18 years were recruited from the LC community rehabilitation service (Leeds, UK) and were invited to take part in the service evaluation if PESE was a significant symptom. They were given written information and completed a consent form and baseline EQ-5D-5L and C19YRSm questionnaires. Both of these outcome measures are used regularly in the service, with the C19YRSm being specific and sensitive to LC symptoms. Those unable to commit to 8 weekly phone calls plus a follow-up call 4 weeks later, and those without sufficient capacity to undertake the study, were excluded.

### 2.1. Pacing Programme

Patients received information and guidance on the WHO Borg CR-10 pacing protocol (Supplementary Materials S1) [25]. This encompasses five incremental phases of activity, alongside a Borg CR-10 rating of perceived exertion (RPE) of 0–10, with 0 being complete rest and 10 being maximal perceived exertion, providing patients with a simple subjective assessment of effort level during an activity. Examples of activities and effort levels were provided to participants.

The pacing protocol helped patients gauge effort levels during activity and assess which phase of activity felt appropriate in the context of symptoms. Patients also identified and introduced an active rest activity, incorporating this into their daily routine (Supplementary Materials S1). The term active rest describes activities aimed at stimulating a parasympathetic response, such as resonant breathing exercises or meditation techniques. To help the adoption of active rest, patients were asked to (if feasible) remain in activity phase two for the first week of the study, meaning their Borg score stayed at no more than 3 out of 10. This, it was hoped, would help patients adopt the notion of restorative, rather than passive, rest.

Patients monitored PESE symptoms in response to the use of the protocol, either progressing, regressing, or remaining at the same phase of activity each week. This was intended to promote autonomy and assist in building confidence to adjust activity levels when necessary, following the principle of remaining as active as possible within the limits of symptoms.

### 2.2. Outcome Measures

Patients completed the Leeds PESE Questionnaire (LPQ), a 4-question Likert scale, (Supplementary Materials S2) and C19YRSm and EQ-5D-5L outcome measures at the start of the programme (baseline). They then engaged in 8 weekly phone calls with a clinician, which included completing the LPQ and Likert scale and discussing any significant issues or events. The C19YRSm and EQ-5D-5L were completed again at week 8, and patients self-managed independently for 4 weeks before a follow-up call at week 12 to complete the LPQ, Likert scale, C19YRSm, and EQ-5D-5L for the final time. Patients were also asked to complete a short qualitative questionnaire to record their experience of this study and the effect of the programme on PESE and LC symptoms.

#### 2.2.1. EQ-5D-5L

The EuroQol EQ-5D-5L is a health-related quality-of-life measure consisting of five domains: mobility, usual activities, self-care, pain/discomfort, and anxiety/depression. Each domain is scored between 1 (no problems) and 5 (severe problems). Responses are collated into a profile score and then converted into a health utility or index score using a country-specific algorithm (tariff or value set). The utility score is measured on a metric from 0 (dead) to 1 (perfect health), reflecting the preferred health status in society for that country [26].

#### 2.2.2. C19-YRSm

The COVID-19 Yorkshire Rehabilitation Scale (C19-YRS) was developed to measure symptoms, functioning, and disability associated with COVID-19, and was the first condition-specific patient-reported outcome measure in the literature. The modified version, C19-YRSm, has 17 items across four sub-scales: symptom severity (0–30), functional disability (0–15), other symptoms (0–25), and overall health (0–10). Each item is scored between 0; no problem and 3; severe problem [27]. Minimal clinical important difference (MCID) has been estimated to be 4 points for the symptom severity subscale and 4 points for the functional disability subscale. Evaluation of the C19-YRSm revealed it is a valid, reliable, and responsive measure [28], hence its inclusion in both our service and this service evaluation.

#### 2.2.3. PESE Characteristics

The standard Leeds PESE Questionnaire (LPQ) recorded the number and nature of PESE episodes over the past 7 days. The Likert scale comprised 3 questions adopted from the Multidimensional Assessment of Interoceptive Awareness (MAIA) V2 2018 [29] and a 4th relating to confidence in completing the diaphragmatic breathing technique. This was included as it related to established practice informed by the literature on resonant breathing [30] and by the HEARTLOC study [31]. The LPQ and Likert scale can be found in Supplementary Materials S2.

### 2.3. Statistical Analysis

Patient age, EQ-5D-5L, and C19-YRSm measures were presented as the mean (SD), duration of LC was presented as the median (IQR), and categorical characteristics as the number of participants in each category (%). Participant characteristics at baseline (week 0) are presented descriptively by intervention group for comparison.

Mixed-effects linear regression was used to compare outcomes at baseline (week 0) with the end of intervention (week 8) and end of follow-up (week 12) for EQ-5D-5L utility scores and visual analogue scales, C19YRSm symptom severity scores, functional disability scores, and overall health scores, and C19YRSm PESE scores, adjusting for age and gender.

Week-on-week changes in weekly process measures (measured weekly during intervention weeks 0 to 8 and at final follow-up week 12) were modelled, adjusting for age and gender and taking into account the serial time measures within each patient. Mixed-effects Poisson regression was used to model the number of PESE episodes per week and the number of symptoms per week, with random slopes over time (weeks 0 to 12), with estimates presented as the percentage change in incidence per week. Mixed-effects linear regression was used to model symptom severity and duration of episodes, activity phase, and active resting score over time, with estimates presented as absolute changes in outcome per week.

The current study is a feasibility study for testing the revised protocol in a cohort of long-term patients. The sample size was a convenience sample based on the participants we could recruit from the service. However, based on data collected as part of an earlier study [25], we estimated that ~70 participants followed-up for 12 weeks would provide 80% power to detect a weekly reduction in the number of PESE episodes of ~3% per week, or 90% power for a reduction of ~3.4% per week, at *p* < 0.05, with similarly good precision for other outcome measures.

## 3. Results

A total of 47 patients were invited to take part in this programme. Three were no longer eligible to participate because their symptoms had improved sufficiently prior to this study, seven could not be contacted or did not return consent, and six were unable to complete because of acute illness. This left a total of 31 patients who received the pacing programme.

Demographic and clinical characteristics at baseline (week 0) are shown in Table 1. The mean (SD) age of participants was 47 (11) years, with more females (65%) than males. Nearly half (45%) of participants were not in full-time paid employment. On clinical measures, the median (IQR) duration of LC symptoms was >2 years (29 months).

### 3.1. Changes in EQ-5D-5L

On completion of the intervention (week 8), there was no evidence of improvement in EQ-5D-5L utility (change = 0.00, 95% CI −0.04 to 0.05, *p* = 0.9) or VAS scores (4, −1 to 9, *p* = 0.1) (8 points, 95% CI 4 to 11) after adjusting for age and gender. At the end of follow-up (week 12), there was no evidence of improvement in EQ-5D-5L utility (0.04, −0.01 to 0.09, *p* = 0.1) but there was evidence of improvement in the EQ-5D-5L visual analogue scale (8 points, 4 to 11, *p* < 0.001) (Table 2).

### 3.2. Changes in C19YRSm

There was evidence of improvements in C19YRSm functional disability (−0.9, −1.7 to −0.2, *p* = 0.01) and C19YRSm PESE subscore (−0.5, −0.7 to −0.2, *p* < 0.001) on completion of the intervention at 8 weeks, and improvement in all C19YRS measures at the 12-week follow-up (Table 2).

### 3.3. Changes in PESE Characteristics

Weekly changes in process measures over time within the intervention group are shown in Supplementary Materials S3 for weeks 0 to 8 of the intervention, then for week 12 after the intervention had been completed. There was evidence of improvement across process measures during the intervention (Table 3), with the number of PESE episodes decreasing gradually each week (15% fewer each week, 95% CI: 11% to 20%, *p* < 0.001), episodes of shorter duration and with fewer symptoms, and episodes of milder severity each week (Table 3). There was no evidence of overall change in the Borg activity phase, reflecting the initial reduction in activity during pacing, before gradual increases over the remaining 8 weeks until activity returned close to its previous levels, whilst maintaining decreased numbers of PESE episodes and symptoms, reduced symptom severity, and shorter duration of episodes. The active resting score also improved over time within the pacing intervention group.

**Figure 1:**
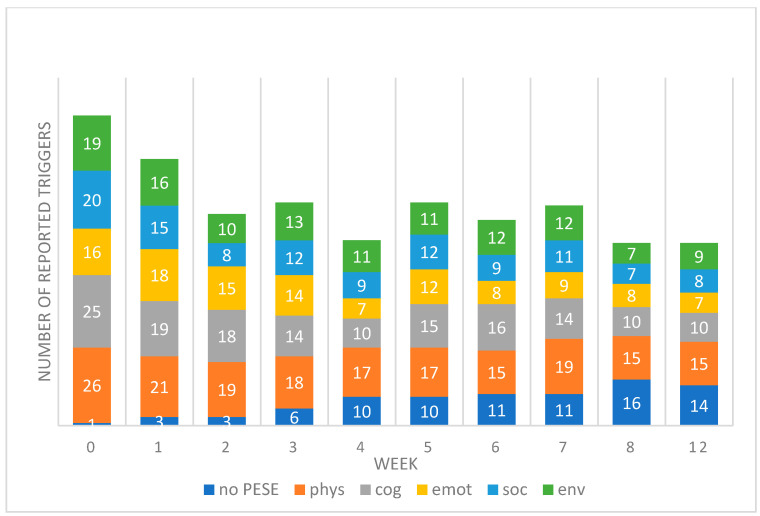
Total number of reported triggers of PESE each week, broken down into each type and including the number of patients reporting no PESE.

The total number of reported triggers of PESE reduced by 54% over the weeks, with a total of 107 reported triggers at week 0 (baseline), 47 at week 8, and 49 at week 12. The number of patients reporting no PESE each week (and therefore no triggers) rose from one (3%) at week 0 (baseline) to sixteen at week 8 (51%) and fourteen (45%) at week 12. A full breakdown of reported triggers per week is given in Figure 1.

**Figure 2:**
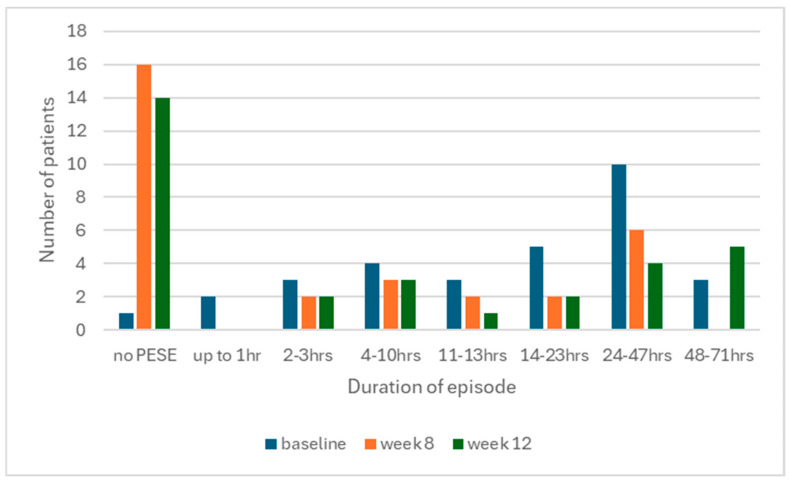
Mean duration of PESE episodes at baseline and week 8.

When PESE did occur, patients reported slightly fewer symptoms with a median of three at baseline to two at week 8, as well as reduced severity, with a median of 2.5 out of 3 at baseline to 1 out of 3 at week 8. We also observed fewer episodes of longer duration by week 8, resulting in a statistically significant improvement overall (Figure 2, Table 3).

**Figure 3:**
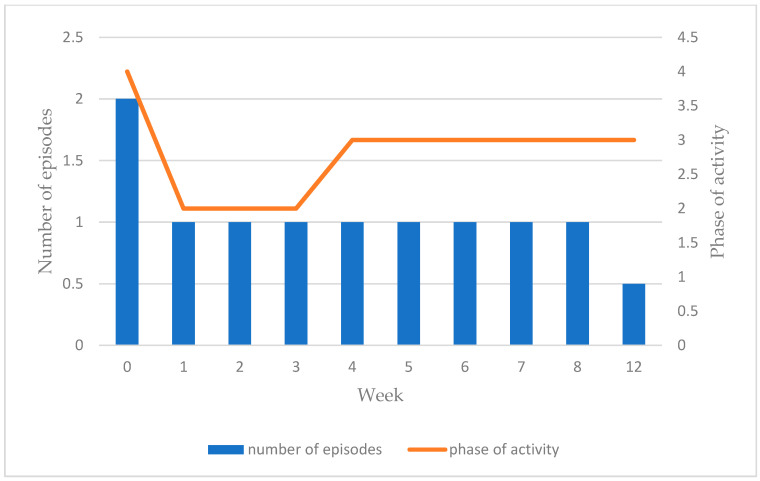
Mean number of PESE episodes and mean phase of activity per week.

Overall, no significant increase in the activity phase was seen from baseline to week 12. However, patients intentionally reduced their phase of activity between baseline and week 1 whilst they began the process of adopting the programme protocol, before gradually re-building them as they felt able. Activity levels steadily returned to near previous levels over the course of the programme, whilst decreased numbers of PESE episodes and symptoms, reduced symptom severity, and shorter durations of episodes were maintained (Figure 3).

Active resting score (Likert) also showed statistically significant changes over time, increasing from a median of 23 at baseline to 30 at weeks 8 (0.7, 0.4, 0.9, *p* < 0.001) and 12 (0.5, 0.3, 0.6, *p* < 0.001). This shows self-reported improvements in both interoceptive awareness and ability to carry out diaphragmatic breathing. The data are enhanced by some of the qualitative data collected at the end of this study (Table 4), though it should be noted it was beyond the scope of this study to perform a full qualitative analysis.

## 4. Discussion

Our service evaluation demonstrates that use of the WHO Borg CR10 pacing protocol over 8 weeks is associated with decreased PESE episodes, a reduction in the number of longer episodes, fewer symptoms, and milder symptom severity. This was reflected in statistically significant improvements in C19YRSm scores and EQ-5D-5L VAS, but the improvements in C19-YRS were not clinically significant. This implies that the changes are not of the degree that the individuals will notice a change in their condition. This reflects the long-term nature of the condition.

The lack of improvement in EQ-5D-5L utility score over the intervention period is in contrast to our earlier work [25], which reported statistically significant improvement across all domains after 6 weeks of using the WHO Borg CR-10 pacing protocol. This may be related to participants with a shorter duration of symptoms (17 months) in our earlier study, compared to the substantially longer duration of LC symptoms in the current study (29 months). We know that a longer duration of symptoms is associated with poorer prognosis in ME/CFS [32], and other authors have reported a lack of full recovery in LC when studied over time [13,14,16,17]. Work by Hastie et al. 2022 [13] and Hurt et al. [14] studied those with a shorter duration of symptoms than in our study (6–18 months, and a mean of 23.2 months, respectively), yet still reported that whilst some improvements were seen over time, full recovery rates were low. In line with these findings, we did not see significant improvements in LC as a whole but did observe improvements in some measures, which is encouraging, despite the sample being an uncontrolled cohort. In addition, in this study, it seemed that change continued over the follow-up period; by week 12, there was a greater change in scores. EQ-5D-5L VAS, C19YRSm symptom severity, functional disability, and overall health scores were not statistically significant at baseline to week 8 but were significant at week 12. This suggests a slower pace of change with an increased duration of symptoms, which has implications both for further research and clinical practice.

The data regarding triggers for PESE may offer additional insight into the pace of change in EQ-5D-5L and C19YRSm scores. We asked patients to consider social and environmental triggers, as well as physical, cognitive, and emotional causes, thereby encompassing most aspects of daily life. Many identified several triggers within their episodes, perhaps recognising overlaps and patterns not previously perceived despite how long they had been experiencing symptoms. We wonder whether the impact of PESE as a stubborn and complex symptom, present for over two years, was overwhelming, and that this provoked a negative response initially. This would align with previous work showing increased symptom burden and psychological distress in ME/CFS patients who experience PESE [33,34].

We believe that there is an implication for practice when comparing the differences in EQ-5D-5L and C19YRSm scores in our study to those of our previous work [25]. Specifically, the WHO Borg CR10 pacing protocol may be most effective when implemented as early as possible following the onset of symptoms, meaning early referral is key. For those with prolonged symptom durations, we need to recognise that they may now have transitioned to a long-term condition and allow for slower, more gradual change. We would argue that pacing is no less important for this group as it remains a cornerstone of fatigue and PESE management, but we should not expect to see rapid changes. Instead, our focus should be on individualised self-management support for patients, using the protocol to help maximise activity levels (within the context of symptoms), minimise avoidance and fear, and promote quality of life.

Interestingly, median C19YRSm PESE scores were statistically significant by week 8 of the intervention, in contrast to the symptom severity score, which was significant only at 12. This sits alongside statistically significant changes in PESE characteristics throughout the programme, namely improvements in the number of episodes, the number of symptoms, the severity of symptoms, and the duration of episodes. The improvement in Likert scores was also statistically significant by the end of week 8, showing that patients became more interoceptively aware and more confident in practising resonant breathing. We feel it is possible that improvements in interoceptive awareness and time spent on active rest may have helped address the boom–bust cycle when combined with a commitment to manageable effort levels via the use of the Borg scale and activity phases. There are two aspects to this. Firstly, emphasising the importance of balancing activity with quality rest, alongside weekly telephone calls, may have led to greater consistency of activity. In a previous study, activity consistency was associated with reductions in depression and avoidance, as well as increases in function, in a group of chronic pain patients [35]. We feel a similar effect may have been experienced in our group, whereby planning time for active rest and consciously allowing oneself to remain at a manageable effort level resulted in an overall sense of coping more effectively with PESE. In this way, we would hope that the use of the programme over time could lead the way to increased and/or more meaningful activity, rather than focussing on symptom reduction. Secondly, the rest aspect of pacing has previously been described as: sleep, relaxation, inactivity, active restoration, and self-regulation [36]. However, there is often little explanation beyond that, and in the context of PESE, fatigue, and brain fog—all associated LC symptoms—it is possible that some patients do not have the energy to really think about, or engage with, what meaningful rest might mean to them. Our focused work on active rest activities and how to incorporate these into daily routines was generally well-received and the associated changes in Likert scores are encouraging. Overall, we feel that promoting active rest added to the efficacy of the intervention and that further investigation of this concept is warranted. Further work could focus on the use of the protocol, including active rest, in the management of other long-term conditions and also via the use of digital formats. This could be especially poignant in the context of vocational rehabilitation given the number of people whose ability to work has been affected by LC [4]; however, we feel that there is scope for transfer to any activity as part of an individualised multidisciplinary rehabilitation programme.

There are several limitations to our service evaluation, including a small sample size and a lack of diversity in our patient group. We were unable to include a comparable control group, which would have added to the quality of our data greatly. Clinicians were not blinded in any way and we were very conscious of opportunities for bias. That said, we feel there are lessons to learn from our results, not least that taking the time to rest well and maintain manageable activity levels can lead to a reduction in the frequency, impact, and duration of PESE. The intervention was generally easy to implement in terms of clinical time and space and did not require expensive resources. Many patients reported preferring frequent short phone calls to lengthy clinic appointments as it was less burdensome on energy levels and meant that issues could be addressed quickly and easily. For us as a clinical rehabilitation service, these are important observations and we aim to continue developing pacing within our practice.

## 5. Conclusions

PESE has become a hallmark of LC and for many, their symptoms are becoming synonymous with ME/CFS. Effective pacing remains one of the only recommended management strategies for PESE, and although simple in principle, it can be difficult to master. We have made minor refinements to the pacing programme by including active rest and extending our period of observation, with some degree of success. We feel the non-pharmacological nature of pacing and its potential to be used alongside wearable technology is an area of research worth pursuing for both LC and ME/CFS. We need to be mindful that changes to overall reported health outcomes may take time and be small, and that only by measuring change over a prolonged period of time will we have any indication of real impact. For that reason, we would support further and larger studies with robust designs (controlled clinical trials). From a clinical perspective, NHS resources to support long-term conditions are stretched, and people are struggling to maintain their day-to-day activities, especially work. A pacing intervention that can be delivered virtually, in shorter appointments, but which results in real-life tangible changes in quality of life provides a valuable and cost-effective intervention that is easily deployable throughout the LC community, and therefore we advocate its use.

## Figures and Tables

**Table 1:** Demographic and clinical characteristics at baseline: week 0.

Characteristic	(n = 31)
Mean age (SD)(years)	47 (11)
**Gender** Female gender (%)	20 (65%)
**Ethnic group** Asian or Asian British Black, Black British, Caribbean, or African Mixed or Multiple ethnic groups White	3 (10%) 1 (3%) 0 (0%) 27 (87%)
**Employment status** Employed or self-employed Reduced hours or flexible hours Sick leave Unemployed Retired/ill health retirement	17 (55%) 3 (10%) 2 (6%) 3 (10%) 6(19%)
**Co-morbidities** Respiratory condition Hypertension Heart condition Type 2 diabetes Thyroid condition Cancer Osteoarthritis Mental health condition	5 (16%) 5 (16%) 1 (3%) 3 (10%) 1 (3%) 1 (3%) 1 (3%) 11 (35%)
**Median duration of Long COVID symptoms** (IQR)(months)	29
**EuroQol EQ-5D-5L** Mean utility score (SD) Mean visual analogue scale (SD)	0.53 (0.19) 47 (15)
**C19YRSm subscales at baseline week 0** Mean symptom severity score (SD) Mean functional disability score (SD) Mean overall health score (SD)	19 (4) 8 (3) 4 (1)

**Table 2:** Outcome measures at baseline (0 weeks), end of intervention (8 weeks), and final follow-up (12 weeks).

	Week 0 Start of Intervention	Week 8 End of Intervention	Change from Week 0 to 8 * (95% CI)	*p*-Value	Week 12 Final Follow-Up	Change from Week 0 to 12 ** (95% CI)	*p*-Value
**Median EQ-5D-5L utility score (IQR)**	0.56 (0.35, 0.65)	0.62 (0.32, 0.71)	0.00 (−0.04, 0.05)	*p* = 0.9	0.59 (0.49, 0.74)	0.04 (−0.01, 0.09)	*p* = 0.1
**Median EQ-5D-5L visual analogue scale (IQR)**	50 (35, 59)	50 (40, 65)	4 (−1, 9)	*p* = 0.1	55 (40, 70)	8 (4, 11)	*p* < 0.001
**Median C19YRSm symptom severity score (0–30) (IQR)**	19 (17, 21)	18 (13, 21)	−1 (−3, 1)	*p* = 0.06	17 (12, 19)	−3 (−4, −2)	*p* < 0.001
**Median C19YRSm functional disability score (0–15) (IQR)**	8 (5, 11)	6 (4, 10)	−0.9 (−1.7, −0.2)	*p* = 0.01	6 (5, 8)	−1.1 (−1.9, −0.4)	*p* = 0.002
**Median C19YRSm overall health score (0–10) (IQR)**	5 (3, 5)	5 (3, 6)	0.2 (−0.2, 0.6)	*p* = 0.2	5 (4, 6)	0.7 (0.4, 1.1)	*p* < 0.001
Median C19YRSm PESE score (0–3) ** (IQR)	3 (2, 3)	2 (2, 3)	−0.5 (−0.7,−0.2)	*p* < 0.001	2 (1, 3)	−0.7 (−0.9,−0.5)	*p* < 0.001

* Change in outcome from week 0 to week 8, based on mixed-effects regression adjusting for age and gender. ** Change in outcome from week 0 to week 12, based on mixed-effects regression adjusting for age and gender.

**Table 3:** Mean questionnaire scores within the intervention group across time points.

Outcome	Week 0 Start of Intervention	Week 4 Midway	Week 8 End of Intervention	Weekly % Change in Incidence Week 0 to 8 * (95% CI)	*p*-Value	Week 12 Final Follow-Up	Weekly % Change in Incidence Week 0 to 12 * (95% CI)	*p*-Value
**Median number of PESE episodes (IQR)**	2 (2, 3)	1 (0, 1)	0 (0, 1)	−15% (−11, −20)	*p* < 0.001	1 (0, 1)	−13% (−9, −17)	*p* < 0.001
**Median number of symptoms (IQR)**	3 (2, 4)	2 (0, 4)	2 (0, 3)	−10% (−5, −14)	*p* < 0.001	2 (0, 3)	−9% (−5, −13)	*p* < 0.001
				Weekly change Week 0 to 8 ** (95% CI)			Weekly change Week 0 to 12 ** (95% CI)	
**Median symptom severity (IQR)**	2.5 (2, 3)	2 (0, 3)	1 (0, 2)	−0.12 (−0.17, −0.07)	*p* < 0.001	2 (0, 2)	−0.09 (−0.13, −0.05)	*p* < 0.001
**Median duration of episodes (IQR)(hours)**	18.5 (7, 24)	7 (0.5, 24)	2.5 (0.5, 24)	−0.3 (−0.4, −0.2)	*p* < 0.001	2.5 (0.5, 24)	−0.2 (−0.3, −0.1)	*p* < 0.001
**Median phase of activity (IQR)**	4 (3, 5)	3 (2, 3)	3 (2, 4)	0.0 (−0.1, 0.0)	*p* = 0.79	3 (2, 4)	0.0 (0.0, 0.1)	*p* = 0.23
**Median active resting score (0–40)(IQR)**	23 (19, 27)	28 (24, 31)	30 (27, 33)	0.7 (0.4, 0.9)	*p* < 0.001	30 (26, 34)	0.5 (0.3, 0.6)	*p* < 0.001

* Average weekly change from mixed-effects Poisson regression adjusting for age and gender. ** Average weekly change from mixed-effects regression adjusting for age and gender.

**Table 4:** Qualitative feedback highlights. Q6: do you feel active rest has made a difference to your PESE symptoms?

Participant ID Number	Answer
6	Yes, it gives you chance to gather yourself and regain your energy levels.
12	Yes. PESE same in terms of onset and severity, but active rest ’attacks’ the edges of it
22	I think it has. I can use the active rest technique that I chose in a lot of situations, it’s easy for me
	to do and so I think I have less PESE or less severe episodes because I have the.
28	Yes I think so, I’ve found that on some occasions I’ve been able to recover enough after
	doing something requiring exertion in the morning that I’m able to do something in the
	evening rather than just being in bed unable to do anything.
35	Yes, it has helped me better understand and manage my symptoms.
36	Yes—I am conscious of the importance of resting well and more routinely so I don’t overdo
	things and end up regularlyexhausted and in pain. I find I don’t need to rest as long if I do it.

## Data Availability

Anonymised data can be obtained by contacting the corresponding author.

## Supplementary Materials

The following supporting information can be downloaded at: https://www.mdpi.com/article/10.3390/jcm14010097/s1, Supplementary Materials S1: WHO Borg 10-CR pacing protocol, Supplementary Materials S2: Leeds PESE questionnaire and 4-item Likert scale for active resting, Supplementary Materials S3: Intervention process measures across time points.
