# Supplementary material for: A Personalised Pacing and Active Rest Rehabilitation Programme for Post-Exertional Symptom Exacerbation and Health Status in Long COVID (PACELOC): A Prospective Cohort Study
Source: J Clin Med. 2024 Dec 27;14(1):97. doi: 10.3390/jcm14010097 (PMC11722468; doi:10.3390/jcm14010097)
Supplement: Supplementary file 1 [file jcm-14-00097-s001.zip › jcm-3376862-supplementary.pdf]

Supplementary material S1: WHO Borg CR-10 pacing protocol

Phase	RPE (0-10)	Example activities
1 – preparation for return to activity	0-1	Diaphragmatic breathing exercises, gentle stretches, Yoga Nidra practice, short walks
2 – low intensity activity	2-3	Walking, light household or gardening tasks,
3 – moderate intensity activity	4-5	Brisk walking, introducing inclines, gentle jogging, resistance exercises
4 – high intensity exercise	5-7	Running, cycling, swimming, dancing
5 – return to baseline	8-10	Usual exercise, sports or daily activities with usual routine and vigour.

Supplemental Material S2: Leeds PESE Questionnaire and 4-item Likert scale for active resting **PESE Service Evaluation Patient Questionnaire**

Initials-

Date completed-

Activity Goal-

Week questionnaire completed (1-8)-

Q1) Have you managed to successfully follow the pacing advice over the past 7 days? *This means you have been able to follow the guidance 7/7 days.*<br>*State which activity phase has been followed (1-5)-*	YES / NO<br>Phase of activity-
Q1b) If no, could you please identify the reason(s) why:	
Q2a) Have you managed to practice restful activity over the past 7 days? *This means you have been able to follow the guidance 7/7 days.*	YES / NO<br>Restful activity-
Q2b) If no, could you please identify the reason(s) why:	
Q3a) Have you experienced any post-exertional symptom exacerbation (PESE) episodes over the past 7 days?	YES / NO
Q3b) If yes, can you please state how many PESE episodes you have experienced over the past 7 days:	
Q3c) If yes, was this triggered by ***physical*** exertion over the past 7 days?	YES / NO
Q3d) If yes, was this triggered by ***cognitive exertion*** over the past 7 days?	YES / NO
Q3e) If yes, was this triggered by ***emotional*** *stress* over the past 7 days?	YES / NO
Q3f) If yes, was this triggered by ***social*** *stress* over the past 7 days?	YES / NO
Q3g) If yes, was this triggered by ***environmental*** stress over the past 7 days?	YES / NO

Q4a) Have your normal activities of daily living (ADLs)- self care, work, rest and play, been affected by PESE over the past 7 days?	YES / NO
Q4b) If yes to the above, how have your ADL – (self care, work, rest and play) been affected?	

Q5) If you have experienced PESE over the past 7 days, how long have your symptoms typically lasted for?	<1 hr	2-3 hrs	4-10 hrs	11-13rs	14-23hrs	≥24 hrs	If >24hr how long:

Please list any symptoms you have experienced during a PESE episode(s) over the past 7 days and rate the severity on a 0-3 scale.

Note: If no PESE symptoms experienced, progress on to next activity phase if suitable.

**Symptom and consequence (following PESE episode)**	**Severity (0-3):**<br>0= None; no problem<br>1= Mild problem; does not affect daily life<br>2 = Moderate problem; affects daily life to a certain extent<br>3 = Severe problem; affects all aspects of daily life; life-disturbing
	0 1 2 3
	0 1 2 3
	0 1 2 3
	0 1 2 3
	0 1 2 3
	0 1 2 3
	0 1 2 3

Other comments/ notes-

Likert Scale

Question:
1. Are you able to notice the difference between tense and calm in your body?<br>0 1 2 3 4 5 6 7 8 9 10<br>Not able to notice Able to notice
2. How much does the feeling of pain/discomfort in your body worry you?<br>0 1 2 3 4 5 6 7 8 9 10<br>Worrying Not worrying
3. How able are you in listening to the fatigue cues in your body?<br>0 1 2 3 4 5 6 7 8 9 10<br>Body ignoring Body listening
4. How able are you to consciously breath nasally, softly, and slowly with your diaphragm<br>0 1 2 3 4 5 6 7 8 9 10<br>Unable to control Able to control

Supplmentary material S3: Intervention process measures across time points

Outcome	Week 0<br>Start of intervention	Week 1	Week 2	Week 3	Week 4<br>Midway	Week 5	Week 6	Week 7	Week 8<br>End of intervention		Week 12<br>After intervention
Median number of PESE episodes (IQR)	2 (2,3)	1 (1,2)	1 (1,2)	1 (1,2)	1 (0,1)	1 (0,1)	1 (0,2)	1 (0,1)	0 (0,1)		1 (0,1)
Median number of symptoms (IQR)	3 (2,4)	3 (2,4)	2 (2,3)	3 (2,3)	2 (0,4)	2 (1,3)	2 (1,3)	2 (0,3)	2 (0,3)		2 (0,3)
Median symptom severity (IQR)	2.5 (2,3)	2 (2,2.5)	2 (2,2.5)	2 (2,3)	2 (0,3)	2 (1,2)	2 (2,3)	2 (0,2.5)	1 (0,2)		2 (0,2)
Median duration of episodes (IQR)(hours)	.25 (0,18.5)	.25 (0,12)	.25 (0,18.5)	.25 (0,7)	.25 (0,7)	.25 (0,7)	.25 (0,7)	.25 (0,7)	.25 (0,.5)		.25 (0,2.5)
Median phase of activity (IQR)	4 (3,5)	2 (2,3)	2 (2,3)	2 (2,3)	3 (2,3)	3 (2,3)	3 (2,3)	3 (2,4)	3 (2,4)		3 (2,4)
Median active resting score (0-40)(IQR)	23 (19,27)	28 (24,30)	28 (24,31)	27 (25,31)	28 (24,31)	30 (26,32)	29 (25,33)	30 (27,33)	30 (27,33)		30 (26,34)
